# Comparative Cytotoxicity Study of PM2.5 and TSP Collected from Urban Areas

**DOI:** 10.3390/toxics9070167

**Published:** 2021-07-14

**Authors:** Ilseob Shim, Woong Kim, Haewon Kim, Yeon-Mi Lim, Hyejung Shin, Kwang Su Park, Seok Min Yu, Young Hee Kim, Hwa Kyung Sung, Ig-Chun Eom, Pilje Kim, Seung-Do Yu

**Affiliations:** 1 Environmental Health Research Department, National Institute of Environmental Research, Incheon 404-708, Korea; wkim416@korea.kr (W.K.); hwkim91@korea.kr (H.K.); ymlim@korea.kr (Y.-M.L.); kwang_su@korea.kr (K.S.P.); show6949@korea.kr (S.M.Y.); heek89@korea.kr (Y.H.K.); optimism3@korea.kr (H.K.S.); iceom@korea.kr (I.-C.E.); newchem@korea.kr (P.K.); sdyu@korea.kr (S.-D.Y.); 2 Climate and Air Quality Research Department, National Institute of Environmental Research, Incheon 404-708, Korea; shjoung@korea.kr

**Keywords:** human alveolar epithelial A549 cell, cytotoxicity, Kelch-like ECH-associated protein 1, nuclear factor erythroid 2-related factor 2, oxidative stress

## Abstract

Ambient particulate matter 2.5 (PM2.5) and total suspended particles (TSPs) are common airborne pollutants that cause respiratory and cardiovascular diseases. We investigated the differences of cytotoxicity and mechanism between PM2.5 and TSP activity in human alveolar epithelial A549 cells. Atmospheric samples from the central district of Seoul were collected and their chemical compositions were analyzed by inductively-coupled plasma mass spectrometry and ion chromatography. PM2.5 and TSP contained high concentrations of heavy metals (Cu, Fe, Zn, and Pb). The most abundant ions in PM2.5 were SO_4_^2−^, NH_4_^+^, and NO_3_^−^. A549 cells were exposed to PM2.5 and TSP (25–200 µg/mL) for 24 h. TSP was more cytotoxic than PM2.5 per unit mass. PM2.5 induced oxidative stress, as evidenced by increased levels of a glutamate-cysteine ligase modifier, whereas low-concentration TSP increased hemeoxygenase-1 levels. PM2.5 and TSP did not affect c-Jun N-terminal kinase expression. The levels of nuclear factor erythroid 2-related factor 2 (Nrf2) in PM2.5- and TSP-treated cells decreased significantly in the cytosol and increased in the nucleus. Thus, Nrf2 may be a key transcription factor for detoxifying environmental airborne particles in A549 cells. TSP and PM2.5 could activate the protective Kelch-like ECH-associated protein 1/Nrf2 pathway in A549 cells.

## 1. Introduction

Particulate airborne pollutants increase the risk of respiratory diseases (e.g., asthma) and cardiovascular diseases (e.g., myocardial infarction and coronary heart disease) in humans through oxidative stress and inflammation [1,2,3,4]. Particulate matter (PM), a well-known risk factor to human health, could exacerbate chronic obstructive pulmonary disease, and even cause lung cancer [5,6,7,8]. Therefore, PM emission is a serious environmental concern, especially in densely populated cities in developing countries that have been affected by vehicular exhaust pollution for many decades.

Total suspended particle (TSP) refers to particles less than 100 µm in size and airborne fine particulate matter (PM2.5) are particles with an aerodynamic diameter of less than 2.5 μm [9]. TSP and PM2.5 are composed of numerous chemical constituents, including heavy metals such as Cu, Fe, Zn, Pb, and As. The concentrations of these metals depend on the source of the pollutant, such as vehicular and industrial emission, fossil fuel combustion for heating or electricity, or waste incineration. Various heavy metals can generate reactive oxygen species (ROS), which can alter the cellular reduction-oxidation state, even at low levels of exposure, and cause toxicity to plants, animals, and humans [10,11,12,13]. Therefore, ambient air-related adverse health effects, such as airway inflammation, allergy, and asthma, might result from oxidative stress caused by an imbalance between the generation of ROS and antioxidant defenses, which results in deleterious effects on cells, and subsequently leads to various diseases or aging [14,15,16,17,18]. Different antioxidant enzymes, such as superoxide dismutase, catalase, glutathione peroxidase, and peroxiredoxin play critical roles against ROS in biological systems [19]. Glutathione (GSH) is a thiol-containing tripeptide that participates in scavenging various xenobiotic electrophiles and glutamate-cysteine ligase (GCS) mediates its synthesis [20,21].

The covalent modification of the highly reactive cysteine residues in Kelch-like ECH-associated protein 1 (Keap1) with electrophilic chemicals allows Keap1 to dissociate from the nuclear factor erythroid 2-related factor 2 (Nrf2) transcriptional regulator, which then accumulates in the nucleus and upregulates the levels of antioxidant proteins, such as hemeoxygenase-1 (HO-1) and the GSH-synthesizing protein, GCS [22,23]. The HO-1 protein is widely studied as a model of redox regulation in mammalian cells and is a useful marker for cellular oxidative stress [15]. Many studies have shown that the Nrf2-antioxidant response pathway plays an essential role against oxidative stress induced by PM [18,24,25]. Nrf2 plays a key role not only in the cellular defense system by stimulating antioxidant genes but also in modulating cell differentiation, protecting against inflammatory lesions, and detoxifying environmental electrophiles, such as aromatic hydrocarbon quinones, crotonaldehyde, acrylamide, methylmercury, and cadmium [26,27,28]. Furthermore, Nrf2 reduces cognitive impairment in Alzheimer’s disease by suppressing oxidation stress and neuroinflammation [29]. In addition, Nrf2 stimulates the upregulation of antioxidant genes, such as HO-1 and superoxide dismutase 1, and induces phase II metabolizing enzymes, including glutathione-S-transferases [30,31,32].

Several studies have reported the inflammation- and oxidative stress-induced cytotoxicity of PM2.5 and PM10 [33,34]. However, to the best of our knowledge, no study has been conducted to compare the cellular damage caused by PM2.5 and TSP collected from urban areas. In addition, the effects of PM2.5 and TSP on the Keap1-Nrf2 detoxification pathway has not been examined. We hypothesized that there is a difference in the cytotoxicity and mechanism of action of PM2.5 and TSP, depending on the size of particles. The objective of this study was to investigate the cellular effect, including the activation of Nrf2, in A549 cells after exposure to TSP and PM2.5. We analyzed the chemical composition of TSP and PM2.5, performed cytotoxicity testing, and investigated the upregulation of the antioxidant-related proteins in human lung epithelial cells, A549.

## 2. Materials and Methods

Ambient PM2.5 and TSP samples were collected from two different monitoring stations located in central Seoul, the Republic of Korea from July to August 2016. Both sampling sites were located in mixed residential and commercial areas and were approximately 7 km apart in a straight line. PM2.5 was collected from Heyhwa, Seoul, using a high-volume air sampler (HV-1000R, Sibata, Saitama, Japan), adapted with a PM2.5 impactor (Sibata). For sampling PM2.5, a quartz microfiber Whatman filter (203 mm × 254 mm × 2.2 µm, Merck, Darmstadt, Germany) was used and pre-combusted at 700 °C for 2 h. The collected samples were stored in a desiccator for 1 day and weighed. TSP was collected from Bulgwang, Seoul, using a high-volume air sampler (TE-5170X, Tisch Environmental, Cleves, OH, USA), which operated at a flow rate of 950 LPM with a quartz microfiber filter (203 mm × 254 mm, Pall Corp., Show Low, AZ, USA), which was baked at 550 °C for 8 h before analysis.

To analyze the heavy metal content in the samples, 5 mL of mixed acid (HNO_3_:HClO_4_ = 4:1) and 1 mL of HF were added to the samples. The resultant mixture was decomposed at 175 °C for 24 h in a graphite block cracker, and the remaining acid was completely volatilized. Thereafter, 2 mL of HNO_3_ (with 0.5 mL of HF) was added to decompose the samples at the same temperature for 24 h, and the remaining acid was completely volatilized. After the dilution of the final digested sample with 20 mL of 1% HNO_3_, the supernatant was separated by centrifugation at 3000 rpm for 30 min (Combi-514R, Hanil Science Industrial Co., Incheon, Korea) and analyzed by inductively coupled plasma mass spectrometry (PerkinElmer, Shelton, CT, USA). For ion analysis, 15 mL of distilled water was added to the collected sample, which was eluted using ultrasonic waves for 30 min and shaken for 1 h. The pre-treated samples were then analyzed using ion chromatography (Metrohm, Herisau, Switzerland) for the quantification of anions and cations.

The morphological and size analyses of PM2.5 and TSP were performed by energy-filtering transmission electron microscopy (EF-TEM, LIBRA 120, Carl Zeiss, Germany). The suspension was filtered through the Advantac paper (0.45-µm pore, cellulose acetate, Cole-Parmer, Vernon Hills, IL, USA). The particles were filtered dropwise through carbon film-coated 300 mesh Cu grids (EMS Inc., Hatfield, PA, USA) and air-dried in a desiccator. Particle size was measured using ImageJ software (NIH, Rockville Pike, MD, USA).

The collected filters were placed in a tube containing distilled water. The filters were subjected to physical impact using a vortex mixer, followed by sonication for 15 min to separate the solid components. An autoclave was used for sterilization of the collected particles. After sterilization, the detached particles were fully dried at 50 °C in an electric heater and weighed. Human lung epithelial cells A549 were obtained from the American Type Culture Collection. The cells were cultured in RPMI 1640 (Thermo Fisher Scientific Inc., Waltham, MA, USA) culture medium (supplemented with 10% fetal bovine serum and 1% penicillin–streptomycin) in an incubator at 37 °C containing 5% CO_2_.

To evaluate the cytotoxicity of PM2.5 and TSP, A549 cells were placed in 96-well plates and pre-cultured for 24 h. Then, the cells were incubated with PM2.5 and TSP (at final concentrations of 0, 25, 50, 100, 200, and 400 µg/mL) for 24 h. Cell morphology was observed by optical microscopy, and cell viability was determined by the water-soluble tetrazolium (WST) assay using EZ-Cytox staining (DoGenBio Co., Seoul, Korea). The clonogenic assay was performed to observe cell growth and determine the colony-forming and cell division ability of the cells. The cells were placed in 6-well plates (200 cells/well) and incubated for 24 h. Next, the cells were treated with PM2.5 and TSP (at the final concentrations of 0, 25, 50, 100, 200, and 400 µg/mL). After 10 days of incubation, the medium was removed, the plates were washed with phosphate-buffered saline (PBS), fixed using methanol for 10 min on ice, and stained by 0.5% crystal violet solution. The number of colonies was counted under a microscope (Olympus Co., Tokyo, Japan).

For each treatment group, A549 cells were washed in PBS and lysed in the Ez RIPA lysis kit (ATTO, Tokyo, Japan). Cytoplasmic and nuclear extracts were prepared using the NE-PER kit (Thermo Fisher Scientific, Rockford, IL, USA), according to the manufacturer’s manual. Proteins were separated by sodium dodecyl sulfate-polyacrylamide gel electrophoresis and were transferred onto a nitrocellulose membrane. After blocking, the blots reacted with primary antibodies against c-Jun N-terminal kinase (JNK), glutamate-cysteine ligase modifier (GCSm), HO-1, Keap1, and Nrf2 for 1 h. Subsequently, they were thoroughly washed thrice with PBS-Tween and incubated with horseradish peroxidase-conjugated secondary antibodies (Santa Cruz Biotechnology, Santa Cruz, CA, USA). Antibody binding was visualized by enhanced chemiluminescence (ATTO), according to the manufacturer’s protocol. Immunoreactive proteins were detected using the Chemiluminescent Imaging System. The expression levels of proteins were normalized using glyceraldehyde 3-phosphate dehydrogenase or lamin B (Santa Cruz Biotechnology).

All data are presented as mean ± standard deviation (SD) and statistical analyses were performed using SPSS version 12 (SPSS Inc., Chicago, IL, USA). One-way analysis of variance (ANOVA) was performed, followed by Student’s *t*-test to compare the exposure groups with the control group. A difference with a *p* value of less than 0.05, 0.01, or 0.001 was considered statistically significant. Each experiment was conducted at least in triplicate. Error bars are used to indicate SD.

## 3. Results

### 3.1. Analysis of PM2.5 and TSP

**Figure 1:**
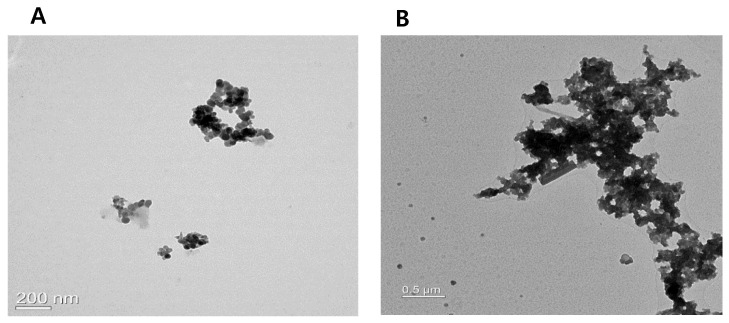
Transmission electron microscopy images of PM2.5 (**A**) and TSP (**B**). Length of bar at lower left section in each photo: (**A**) 200 nm and (**B**) 0.5 µm.

PM2.5 appeared as grape-like clusters consisting of spherical particles, whereas TSP particles existed in both spherical and rod forms (Figure 1).

In addition, heavy metal analysis indicated that the concentrations of Cu, Fe, Zn, and Pb per unit air volume were higher in TSP than in PM2.5 (Table 1).

The concentrations of metals in TSP were in the order of Cu > Al > Fe > Zn > Pb > As > Cr > Cd. The inorganic water-soluble ion analysis showed that SO4^2−^ (8.091 μg/m^3^), NO_3_^−^ (0.662 μg/m^3^), and NH_4_^+^ (2.873 μg/m^3^) were the most abundant ions in PM2.5. SO_4_^2−^ (7.993 μg/m^3^), NO_3_^−^ (2.319 μg/m^3^), and NH_4_^+^ (1.872 μg/m^3^) were also abundant in TSP (Table 2).

**Figure 2:**
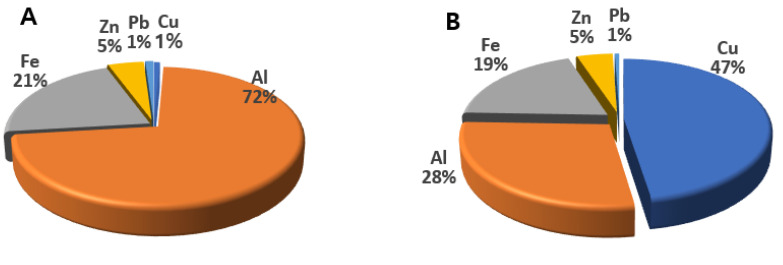
Proportion of metals in PM2.5 (**A**) and TSP (**B**).

**Figure 3:**
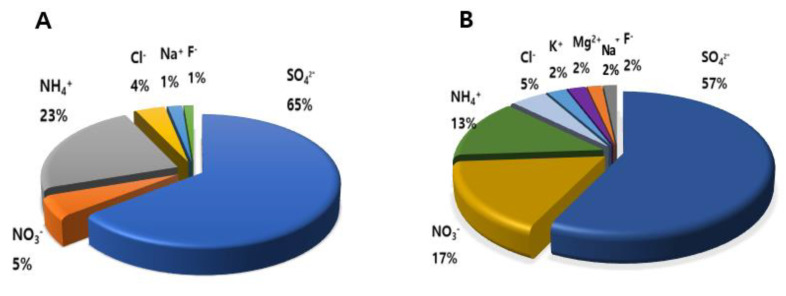
Proportion of ions in PM2.5 (**A**) and TSP (**B**).

Thus, SO_4_^2−^, NO_3_^−^, and NH_4_^+^ were the three major ions in both PM2.5 and TSP, accounting for 93.3% and 87.3% of the total water-soluble ions mass concentration, respectively. The concentration ratio of metals and ions in PM2.5 and TSP was shown in Figure 2 and Figure 3.

Al was the major metal in PM2.5, whereas Cu was the dominant metal in TSP. SO_4_^2−^ accounted for more than half of all ions in PM2.5 and TSP.

### 3.2. In Vitro Assay

**Figure 4:**
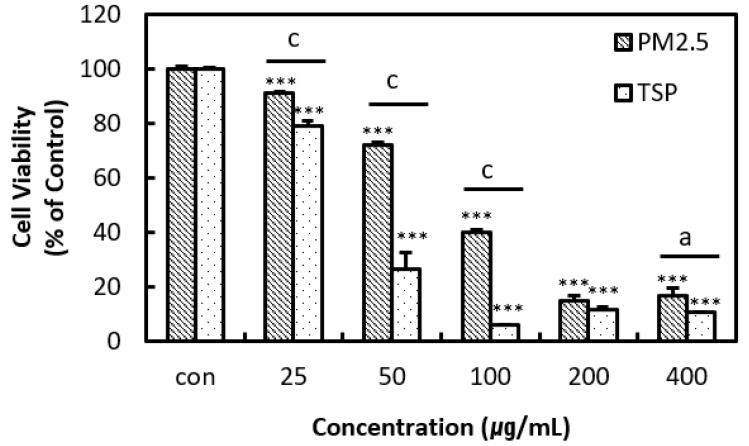
Cell viability measured by the water-soluble tetrazolium assay. Human lung epithelial cells (A549) were treated with PM2.5 and TSP at final concentrations of 0, 25, 50, 100, 200, and 400 µg/mL for 24 h. Experiments were repeated thrice. The values are reported as the mean ± SD (Student’s *t*-test, *** *p* < 0.001, vs. control; ^a^ *p* < 0.05, ^c^ *p* < 0.001).

A dose-dependent statistically significant decrease in cell viability was observed at PM2.5 and TSP concentrations greater than 25 μg/mL. There was a dramatic decrease in the viability of TSP-treated cells at concentrations greater than 50 μg/mL. The IC50 values for PM2.5 and TSP were 73.14 and 47.05 μg/mL, respectively (Figure 4).

**Figure 5:**
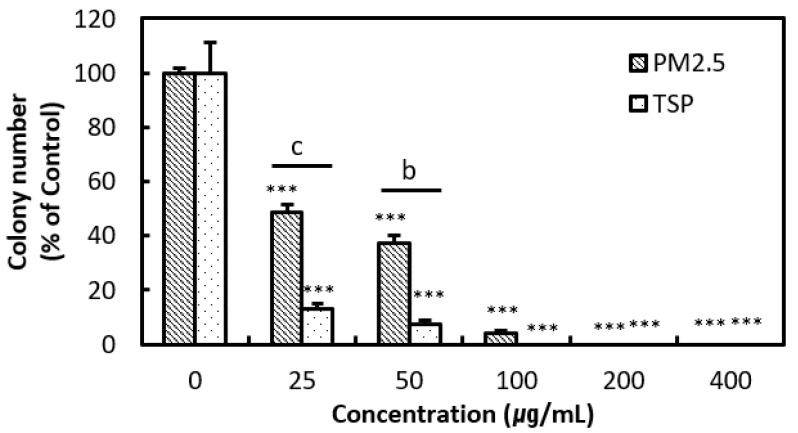
The inhibition of cell colony formation induced by PM2.5 and TSP in human lung epithelial cells (A549). Cells were exposed to PM2.5 and TSP at concentrations of 0, 25, 50, 100, 200, and 400 µg/mL for 10 days. Experiments were repeated thrice. The values are reported as the mean ± SD (Student’s *t*-test, *** *p* < 0.001, vs. control; ^b^ *p* < 0.01, ^c^ *p* < 0.001).

The clonogenic assay was used to evaluate the chronic effect of treatment with PM2.5 and TSP on A549 cells. After 10 days of incubation, the total number of colonies decreased markedly in both PM2.5- and TSP-treated cells as the concentration of PM2.5 and TSP increased to more than 25 μg/mL (Figure 5). There were almost no colonies in TSP-treated cells at concentrations greater than 100 μg/mL. Overall, the cytotoxicity of TSP against A549 cells was higher than that of PM2.5, as observed in the WST and clonogenic assays.

### 3.3. Oxidative Stress-Related Proteins

**Figure 6:**
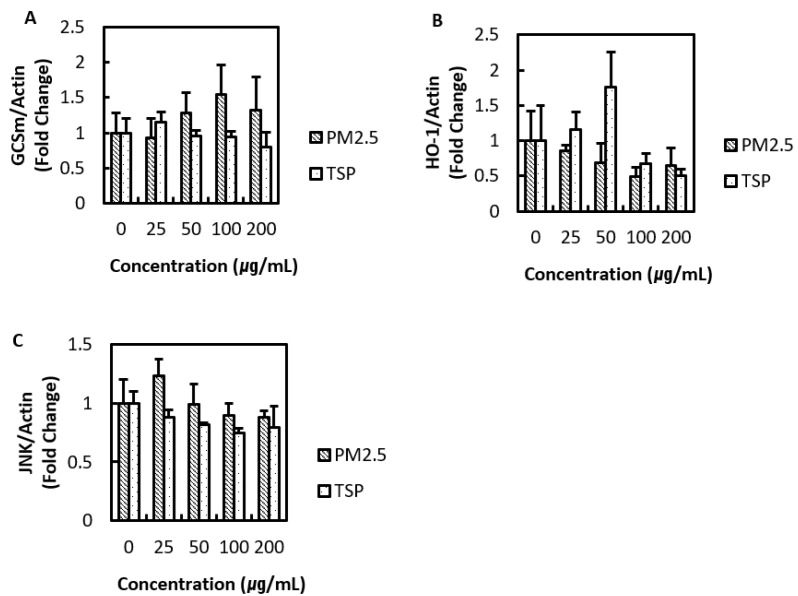
Effects of PM and TSP on the level of the glutamate-cysteine ligase modifier (GCSm), Heme oxygenase 1 (HO-1), and c-Jun N-terminal kinase (JNK). Protein expression of (**A**) GCSm, (**B**) HO-1, and (**C**) JNK were measured in A549 cells exposed to PM2.5 and TSP at 0, 25, 50, 100, and 200 µg/mL for 24 h.

A number of antioxidant proteins are activated in response to oxidative stress in cells. We evaluated the regulation of HO-1, GCSm, and JNK to identify the antioxidant activity of cells against PM2.5 and TSP. PM2.5 increased the oxidative stress in cells, as indicated by increased GCSm protein levels, and lower concentrations of TSP caused an increase in HO-1 levels (Figure 6).

It has been reported that JNK, a mitogen-activated protein kinase, is regulated by various oxidants [35]. However, there was no specific change in JNK levels in A549 cells exposed to PM2.5 and TSP. After band intensity analysis, the expression level of each protein was normalized with the intensity of actin. In summary, exposure of A549 cells to PM2.5 and TSP resulted in the upregulation of antioxidant proteins.

### 3.4. Effects of PM2.5 and TSP on the Keap1-Nrf2 Signaling Pathway

**Figure 7:**
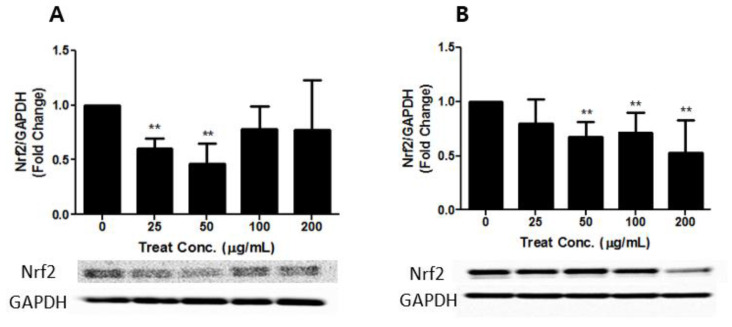
Activation of Nrf2 in cytosol of A549 cells after treatment with PM2.5 (**A**) and TSP (**B**). Cells were exposed to PM2.5 and TSP at 0, 25, 50, 100, and 200 µg/mL for 24 h. The values are reported as the mean ± SD (Student’s *t*-test, ** *p* < 0.01, vs. control).

**Figure 8:**
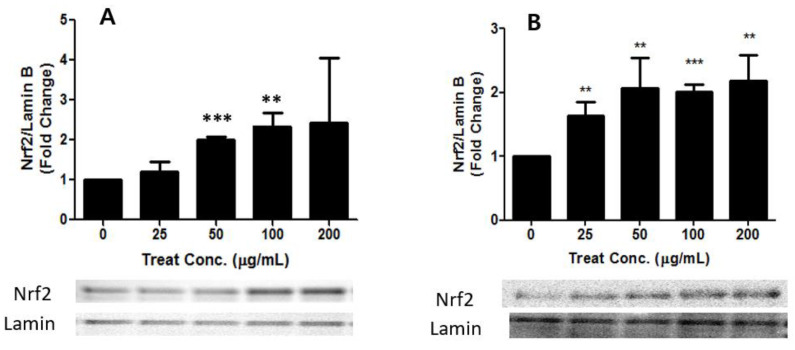
Activation of Nrf2 in nucleus of A549 cells after treatment with PM2.5 (**A**) and TSP (**B**). Cells were exposed to PM2.5 and TSP at 0, 25, 50, 100, and 200 µg/mL for 24 h. The values are reported as the mean ± SD (Student’s *t*-test, ** *p* < 0.01, *** *p* < 0.001, vs. control).

Heavy metals contained in PM2.5 and TSP can catalyze the generation of ROS in the body [36,37], thus, PM2.5 and TSP may lead to the activation of Nrf2 in the in vitro system. Our data demonstrated a dose-dependent increase in the expression of nuclear Nrf2, both in PM2.5- and in TSP-treated cells. The PM2.5- and TSP-treated groups showed a two-fold increase in the levels of Nrf2 in the nucleus compared to the control group. In addition, the expression level of the cytosolic Nrf2 protein decreased both in PM2.5- and TSP-treated cells (*p* < 0.01) (Figure 7 and Figure 8). The activation of Nrf2 in TSP-treated cells was higher than that in PM2.5-treated cells. A similar pattern of results was obtained in our study, which demonstrated a dose-dependent increase in the expression of nuclear Nrf2 in both PM2.5- and TSP-treated cells. In addition, the expression level of the cytosolic Nrf2 protein in PM2.5- and TSP-treated cells showed a statistically significant decrease (Figure 7 and Figure 8).

These results could be attributed to the effects of oxidative stress caused by significant concentrations of heavy metals in PM2.5 and TSP. Variation in the levels of Nrf2 in TSP-treated cells was more prominent than that in PM2.5-treated cells. Our study was carried out with only A549 cells and limited antioxidant proteins, therefore, additional studies, focusing on the toxic effects of PM2.5 and TSP in other in vitro systems and experimental animals, are suggested. Furthermore, biomarker-based studies would be useful to evaluate and compare the oxidative stress and redox activity of PM2.5 and TSP.

## 4. Discussion

The chemical compositions of ambient PM2.5 and TSP are considerably complex and diverse, and depend on sampling time, region, and a natural or anthropogenic source [30,31,32]. Our data (Table 1) showed that both ambient TSP and PM2.5 contained different types of heavy metals, such as Cu, Pb, As, and Cd. Our data on the concentration of heavy metals were similar to those obtained from a previous study [30,31,32], however, the proportion of metal was different between PM2.5 and TSP, which might be attributable to the different sampling sites. The most abundant water-soluble ions in TSP and PM2.5 were SO_4_^2−^, NO_3_^−^, and NH_4_^+^. In addition, SO_4_^2−^ and NH_4_^+^ were more abundant in PM2.5 than in TSP, whereas NO_3_^−^, Cl^−^, K^+^, Mg^2+^, Na^+^, and F^−^ were more abundant in TSP.

Both TSP and PM2.5 produced significant cytotoxicity in A549 cells in a dose-dependent manner. However, TSP was significantly more cytotoxic than PM2.5. In a study conducted by Deng et al., A549 cells were exposed to 25, 50, 100, and 200 μg/mL of PM2.5 for different durations [30,31,32]. Although several studies have investigated the cytotoxicity of PM2.5, data regarding the cytotoxic potential of TSP is limited [38]. PM2.5 and ultrafine particles (<0.1 μm) can penetrate the alveoli of the lungs and enter the blood circulation, ultimately causing cardiovascular morbidity in humans [39,40]. On the other hand, PM10 deposits mainly in the head region owing to its larger size, and is considered less dangerous than PM2.5 or PM0.1 [41]. Therefore, greater attention has been focused on the more hazardous PM2.5 when it comes to human health risk. Our study revealed that TSP had higher cytotoxicity than PM2.5, however, these results were obtained in vitro system and could be the main limitation of this study. Differences in cytotoxicity between PM2.5 and TSP might be primarily owing to differences in particle size and retention capabilities or could be dependent on the differences in the respective compositions [6].

Airborne PM is a major component of air pollution and is associated with various diseases, such as cardiovascular and respiratory illness, through the generation of ROS [31,42,43]. Therefore, oxidative stress induced by PM is considered a major mechanism of PM toxicity in humans and animals [44,45,46]. The generation of free radicals owing to airborne PM can induce oxidative stress and the activation of mitogen-activated protein kinase pathway proteins [47]. JNK is a mitogen-activated protein kinase involved in oxidative stress-related cellular responses [48,49,50], such as airway epithelial apoptosis [51]. The antioxidant protein, HO-1, is upregulated when Nrf2 migrates into the nucleus and binds to the antioxidant response element [52,53]. In addition, HO-1 plays an important role in the regulation of apoptosis and modulation of inflammation through catalyzing heme degradation and the generation of iron ions, bilirubin, and carbon monoxide [54]. The Nrf2 signaling pathway induces the elevation of HO-1 and GCSm expression in human umbilical vein endothelial cells [55]. Many studies have suggested that PM2.5 causes oxidative stress in various in vitro systems [34,56,57]. Similar to several previous findings, our results demonstrated that PM2.5 and TSP lead to an obvious increase in oxidative stress. Specifically, exposure to PM2.5 caused an increase in the levels of GCSm in A549 cells, whereas TSP exposure led to an upregulation of HO-1, even at lower concentrations. However, we could not find any specific change in the levels of JNK in A549 cells exposed to PM2.5 and TSP.

Cells are equipped with various defense mechanisms, including stress response pathways, to protect themselves against stressful conditions. The Nrf2-Keap1 signaling pathway is one such stress response pathway that is activated to amend homeostatic imbalance and deactivated when homeostasis is restored. Recent studies have suggested that Nrf2 plays a crucial role not only in cellular defense against intrinsic and extrinsic cellular stresses but also in anticancer drug resistance [58,59]. When reactive oxidants or electrophiles, such as heavy metals, are present in sufficient concentrations in the cells, electrophiles covalently modify Keap1 through the Michael addition, thereby dissociating Nrf2 from Keap1 [60]. Nrf2 also enhances the synthesis and recycling of GSH, which is the most dominant cellular antioxidant. GSH specifically induces enzymes, such as glutamate-cysteine ligase catalytic and modifier subunits and GSH reductase [61]. Li et al. showed that PM2.5 activates the Nrf2 transcription factor via detoxification in cells [62].

## 5. Conclusions

This study was performed to investigate the cellular effect, including the activation of transcription factor Nrf2, in A549 cells after exposure to TSP and PM2.5. Other studies have focused on the respirable PM until now, however, this study has been done to determine whether TSP has a similar effect in vitro system. In summary, our results showed that TSP was more cytotoxic than PM2.5 to A549 cells. We also demonstrated that both PM2.5 and TSP have the potential to activate the cellular protective Keap1/Nrf2 pathway in A549 cells, indicating that both the materials might generate ROS inside cells.

## Figures and Tables

**Table 1:** Concentrations of metal in PM2.5 and TSP.

Elements	PM2.5 (ng/m^3^)	TSP (ng/m^3^)
Cu	9.26 ± 3.15	2572 ± 593
Al	899 ± 11	1520 ± 54
Fe	263 ± 30	1045 ± 32
Zn	57.3 ± 10.7	247.4 ± 88.5
Pb	12.96 ± 1.90	31.05 ± 17.51
As	3.10 ± 1.20	8.10 ± 5.65
Cr	3.89 ± 0.21	7.96 ± 2.52
Cd	0.45 ± 0.04	1.04 ± 0.44

Data shown as mean ± S.D.

**Table 2:** Concentrations of ion in PM2.5 and TSP.

Ions	PM2.5 (μg/m^3^)	TSP (μg/m^3^)
SO_4_^2^^−^	8.091 ± 2.887	7.993 ± 2.102
NO_3_^−^	0.662 ± 0.219	2.319 ± 1.510
NH_4_^+^	2.873 ± 0.920	1.872 ± 0.851
Cl ^−^	0.431 ± 0.003	0.629 ± 0.003
K^+^	N.D.	0.335 ± 0.049
Mg^2+^	N.D.	0.317 ± 0.054
Na^+^	0.243 ± 0.032	0.257 ± 0.048
F^−^	0.162 ± 0.003	0.236 ± 0.003

Data shown as mean ± S.D.; N.D.: Not Detected.
